# Qualitative study of patient experiences of mental distress during TB investigation and treatment in Zambia

**DOI:** 10.1186/s40359-022-00881-x

**Published:** 2022-07-19

**Authors:** T. Mainga, M. Gondwe, I. Mactaggart, R. C. Stewart, K. Shanaube, H. Ayles, V. Bond

**Affiliations:** 1 Zambart, University of Zambia School of Public Health, Ridgeway, Zambia; 2 Department of Global Health and Development, London School of Hygiene and Tropical Medicine, London, UK; 3 Department of Clinical Research, London School of Hygiene and Tropical Medicine, London, UK; 4 Division of Psychiatry, University of Edinburgh, Edinburgh, UK; 5 Malawi Epidemiology and Intervention Research Unit (MEIRU), Lilongwe, Malawi; 6 Department of Department of Population Health, International Centre for Evidence in Disability, London School of Hygiene and Tropical Medicine, London, UK

**Keywords:** Tuberculosis, Mental distress, Contextual drivers, Poverty, Zambia, Patient experiences

## Abstract

**Background:**

The mental health and TB syndemic is a topic that remains under-researched with a significant gap in acknowledging and recognizing patient experiences, particularly in the sub-Saharan African region. In this qualitative study conducted in Zambia, we aimed to explore the lived mental health experiences of TB patients focusing on their multi-layered drivers of distress, and by so doing highlighting contextual factors that influence mental distress in TB patients in this setting.

**Methods:**

The study draws on qualitative data collected in 2018 as part of the Tuberculosis Reduction through Expanded Antiretroviral Treatment and Screening for active TB trial (TREATS) being conducted in Zambia. The data was collected through in-depth interviews with former TB patients (n = 80) from 8 urban communities participating in the TREATS trial. Thematic analysis was conducted. Additional quantitative exploratory analysis mapping mental distress symptoms on demographic, social, economic and TB characteristics of participants was conducted.

**Results:**

Most participants (76%) shared that they had experienced some form of mental distress during their TB investigation and treatment period. The reported symptoms ranged in severity. Some participants reported mild distress that did not disrupt their daily lives or ability to adhere to their TB medication, while other participants reported more severe symptoms of distress, for example, 15% of participants shared that they had suicidal ideation and thoughts of self-harm during their time on treatment. Mental distress was driven by unique interactions between individual, social and health level factors most of which were inextricably linked to poverty. Mental distress caused by individual level drivers such as TB morbidity often abated once participants started feeling better, however social, economic and health system level drivers of distress persisted during and beyond TB treatment.

**Conclusion:**

The findings illustrate that mental distress during TB is driven by multi-layered and intersecting stresses, with the economic stress of poverty often being the most powerful driver. Measures are urgently needed to support TB patients during the investigation and treatment phase, including increased availability of mental health services, better social security safety nets during TB treatment, and interventions targeting TB, HIV and mental health stigma.

*Trial registration* ClinicalTrials.gov NCT03739736. Trial registration date: November 14, 2018.

**Supplementary Information:**

The online version contains supplementary material available at 10.1186/s40359-022-00881-x.

## Background

There is growing global acknowledgment of the relationship between tuberculosis (TB) and mental distress [1–4]. Mental (or psychological) distress refers to “a state of emotional suffering characterized by symptoms of depression and anxiety” that can range from mild and transitory states to long-lasting and severe medically defined disorders [5]. Studies conducted in 2004 and 2009 indicate that the prevalence of common mental health disorders (CMD) in the general population in Zambia is between 20 and 14% respectively [6, 7]. Despite the potentially high prevalence of CMD, there is still a significant shortage of mental health services in country [8].

Prevalence rates of CMD amongst TB patients varies by country. A 2020 global systematic review and meta-analysis, that aimed to summarize the prevalence of depression among 4903 TB patients from seven countries, found a pooled prevalence estimate of 45% from 25 studies [9], which is significantly higher than the estimated global prevalence of depression of 3.8% [10]. The review highlighted a broad range of reported prevalence rates of depression from 80% in Pakistan to 16% in India [9]. Only two of the studies were conducted in countries (Turkey and China) that did not fit the criteria for the World Health Organization (WHO) 2016–2020 high TB burden [11], however the prevalence of depression among TB patients reported in the two studies also fell within the range reported from studies conducted in high burdened countries (China 17% and Turkey 61%) [9]. Further evidence, from a cross-sectional analysis of community-based data from 48 low- and middle-income countries, found that TB patients were 3.5 (low-income) and 3.2 (middle-income) times more likely to be depressed than individuals without TB [12].

Zambia is among the top 20 highest TB burdened countries globally when measured by absolute number of incident TB cases [13]. Despite this considerable TB burden there has been little investigation of the mental health implications of a TB episode in the Zambian setting. Published studies conducted with regards to the intersection between TB and mental health in Zambia indicate a range in the prevalence of CMD in TB patients. One study found worryingly high rates of mental distress in their sample of 231 TB patients, with 30.9% expressing suicidality out of which 3.4% had attempted suicide at least once [14].

Mental distress in TB patients increases the likelihood of negative health outcomes by exacerbating poor health seeking behaviour and ability to adhere to treatment [1, 3, 15]. The relationship between TB and mental distress is reciprocal and acts through complex channels [1]. People experiencing chronic mental distress are at higher risk of developing TB, while some biological and social drivers of TB increase the likelihood of mental distress in TB patients [1, 16]. Furthermore, high levels of mental distress have negative implications for TB treatment outcomes as highlighted by a multi-country study consisting of TB patients from Zambia, South Africa, Zimbabwe and Tanzania which showed that participants with higher levels of mental distress were more than two times more likely to be non-adherent than those with lower levels of distress [15].

TB, like many other life-threatening conditions can be considered an adverse life event due to how the disease can negatively alter an individual’s physical and social state. In low and middle income (LMIC) settings like Zambia, individuals with TB may face an array of social economic adversities during the TB investigation and diagnosis period. For example, poverty can serve as a barrier to adequate TB investigation and treatment in this setting [17] and it is also a documented driver of mental distress [18]. Half of the Zambian population faces multi-dimensional poverty [19] and poverty is directly correlated with the TB epidemic, as documented by a 2013–2014 TB prevalence survey which highlighted higher TB prevalence rates in the lowest wealth category [20]. TB stigma is another social driver of mental distress in TB patients [21, 22]. The prevalence of TB stigma in Zambia is high. For example, 16.5% and 39.1% of the 115 patients in one urban community in Zambia reported experiencing high and moderate stigma, respectively [23]. Therefore, to both understand and address mental distress amongst TB patients, it is important to expand beyond a biomedical framework and examine the role that social context plays.

To our knowledge, the studies exploring the relationship between mental health and TB in the Zambian setting have mainly focused on investigating prevalence rates of CMD in TB patients [14, 24, 25]. This trend of quantifying mental distress levels in TB patients reflects other research in sub-Saharan Africa leaving a significant gap in acknowledging and recognizing patient experiences around the nexus of mental health and TB. Yet there is growing recognition of the importance of understanding patients experience of illness, including how illnesses intersect with patients’ daily lives [26]. Furthermore, many TB patients in LMICs may be facing multiple health challenges and have complex health and social care needs that could undermine TB treatment outcomes if not adequately considered. Patient experiences therefore provide valuable knowledge that can be used to improve quality of health care, including mitigating the impact of contextual factors that may negatively impact the illness experience. This qualitative inquiry is the first study in the Zambian setting that aims to unpack the lived mental health experiences of TB patients focusing on their experienced social drivers of distress, and by so doing highlighting contextual factors that influence mental distress in TB patients in this setting.

## Methods

The study draws on qualitative data collected in 2018 as part of the Tuberculosis Reduction through Expanded Antiretroviral Treatment and Screening for active TB trial (TREATS). TREATS is a follow-on study from the Population Effects of Antiretroviral Therapy to Reduce HIV Transmission (PopART) which was conducted in 21 communities, 12 in Zambia and 9 in South Africa. PopART was a randomised control trial that aimed to investigate the impact of combined community level TB/HIV interventions, including TB screening and counselling services, on HIV incidence. The intervention was delivered by approximately 400 community health workers known as Community HIV care Providers (CHiPs). A description of the full PopART intervention is detailed in a previous publication [27]. TREATS aimed to measure the impact of the PopART intervention on TB incidence and prevalence and is therefore being carried out in the same 12 PopART communities in Zambia.

### Study setting

TREATS study communities were selected based on having TB notification rates greater than 400/100,000 per annum, a high HIV prevalence (ranging from 13 to 25%) and a proximity to a TB diagnostic centre [28, 29]. All eight communities are urban and span across five provinces of Zambia. Common infrastructure includes a government health facility, schools, police stations, churches, market areas and transport depots [30]. Formal employment options are limited and most residents work in the informal sector as casual laborers or vendors trading in goods [30].

The government health facilities in all eight communities provide TB and HIV services. These services are provided in different departments and are coordinated by a by referral system [31]. HIV services are provided at the Antiretroviral Therapy (ART) department, where newly diagnosed HIV patients are enrolled in the national HIV program and also undergo clinical evaluations [31]. Newly diagnosed HIV patients are also tested for TB, most commonly though sputum smear microscopy, and are screened for TB symptoms at every ART visit [31]. All HIV positive patients who are diagnosed with TB are referred to the TB clinic to initiate TB treatment [31]. Individuals with presumptive TB are screened at the TB department for both TB and HIV. Those found to be HIV positive are referred to the ART department. The TB department is often staffed by one or two nurses and lay health workers known as TB treatment supports or peer educators [31]. All those diagnosed with drug susceptible TB are commenced on a six month anti- TB medication course as stipulated by national guidelines [31].

### Data collection

Qualitative data was collected by a social science team consisting of seven field-based research assistants (three men and four women). The research assistants were graduates of development studies [5] and social work [2] and were guided by three experienced social scientists who provided training, de-briefing and field support (first, second and last authors). The social scientists were supported by the third author who is a psychiatrist with extensive experience in the region. The mental health training was conducted as component of a broader training around TB and TB stigma in Zambia and included components focused on: sensitivity to participants’ emotional wellbeing; reflection of personal mental health biases; an understanding of emotional wellbeing; and a description of CMD in the region and how they are interlinked to TB in this context. The broader training included: an overview of qualitative data collection methods; research ethics; an overview of the TB and HIV epidemic in Zambia; and an overview of TB stigma in Zambia. The training took place for 8 h a day over 5 consecutive days.

We conducted in-depth interviews (IDIs) with former TB patients across eight communities, four that received the PopART intervention and four that served as control communities during PopART. The eligibility criteria included: being 18 years and older; starting TB treatment between September 2016 and November 2017; having a TB diagnosis confirmed through sputum smear or gene expert; and a TB treatment outcome of ‘complete’ at the time of data collection.

We aimed to recruit 14 participants in each of the intervention communities who were divided into two groups. Seven were diagnosed through passive case finding and their diagnosis came about by participants going to the clinic without having been screened or referred to the clinic by a CHiP. Seven were diagnosed by CHiPs during the PopArt intervention and their diagnosis was a result of participants being screened by CHiPs as part of the CHiPS home visits during the PopART intervention. We therefore aimed to recruit a total of 56 participants from the intervention communities. In the control communities we aimed to recruit a total of 28 participants, 7 from each of the 4 communities. We believed this sample would provide an in-depth comparative perspective on TB diagnosis experiences between individuals diagnosed in the intervention and control communities. All participants from the control communities were diagnosed through passive case finding. TB patients diagnosed through passive case finding were recruited through the clinic TB registers while those diagnosed as part of the PopART intervention were recruited through the CHiPs PopART database. In each of the respective databases we selected the first two names from each month starting from September 2016 inclusive of December 2017 cumulating to 32 potential participants from each site. We used purposive sampling, based on Patton’s maximum variation, to ensure a good distribution of age and sex [32]. Participants were invited to take part in the study through a phone using the cell phone numbers provided in the databases. If we were unable to reach the potential participant or if they declined to take part in the study, then we called the next potential participant who matched the demographic profile of the potential participant we initially tried to locate or invite. In total, 80 individuals agreed to take part in the study, of whom 52 and 28 were from the intervention control communities respectively. We were able to reach data saturation with this sample.

Interviews lasted between 45 min to 1.5 h. The time of the interview was determined by the participants depth of responses to the questions in the interview guide and any other additional information that they were willing to share. Areas of inquiry included an understanding of mental health, experiences and causes of mental distress during TB diagnosis and treatment, the implications of poor mental health for TB patients’ quality of life and TB treatment outcomes, and availability and accessibility of mental health services for TB patients. A semi structured interview guide (see Additional file 1) was used, with probes where appropriate. Topic guides included: introductory questions; perceptions around TB transmission; treatment seeking pathways; TB treatment; impact of TB; TB stigma; support during TB investigation and treatment; TB treatment and food consumption; TB/HIV co-infection; and closing remarks with an invitation for questions and comments from participant. The interview guide was adapted from the Converging Impact of Tuberculosis, HIV/AIDS, and Food Insecurity (RENEWAL) study, which was an anthropological study conducted in rural Zambia and peri-urban South Africa between 2006/7. The RENEWAL study documented the impact of TB and HIV co-infection in context of poverty and overstretched public health services and was conducted in 18 households affected by TB and in 17 comparative non-affected households. Findings from the RENEWAL study have been published previously [17, 32].

Research Assistants and participants agreed on the most convenient and private locations to conduct the interviews; these were often in participants homes (living rooms or an outside sitting area) or in a private location at a participating government clinic closest to the participant’s home.

### Ethical considerations

Ethical approval for all study procedures was obtained from the Observational Research Ethics Committee of the London School of Hygiene and Tropical Medicine (LSHTM) (#14985), and the Bio-medical Ethics Committee of the University of Zambia (005/02/18). Informed written consent was obtained from all participants prior to data collection activities. The emotional wellbeing of participants was central to the research process and participants were welcome to revoke participation at any point during the interview.

### Data analysis

Interviews were conducted and recorded either in English or a local language that the participant was comfortable with. English transcripts were transcribed verbatim, while those conducted in local languages were translated into English during transcription. Data was analyzed thematically using a coding framework developed around the TB mental health literature, and the WHO International Classification of Functional disability and health framework. Further codes were added during analysis. Data was double coded by the first and second authors using ATLAS ti software. Any discrepancies with themes emerging from the data were resolved by correspondence with the last author and securing common consent among the first, second and last authors. Additional exploratory analysis of how demographic, social, economic and TB characteristics (including TB history) mapped on mental distress symptoms of participants was conducted in excel by the first author and reviewed by the last.

For this analysis *TB stigma* is defined as a dynamic social process that is enacted through individuals, communities, organisations, and structures [33] resulting in the devaluation of people with TB [34]. It manifests as; experiences of exclusion and/or discrimination; perception, expectation and/or fear of being stigmatized; and feelings fear and/or shame and a loss self-esteem and dignity [35], due to confirmed or suspected TB disease. *Social isolation* is defined as the process of being separated from normal social networks due to TB. *Negative attitude* towards people with TB is defined as being rude, aggressive, and/or ignoring people with suspected of confirmed TB.

## Results

The results firstly provide a summary of mental distress characteristics of participants that are then presented under three broad themes as identified by the analysis. The first theme examines individual level drivers of mental distress, while the second theme is focused on social and economic household level drivers of distress and the last theme examines health systems level drivers of mental distress as experienced by participants during TB investigation and treatment. Table 1 below, provides a description of participant characteristics. All the participants in the study had drug sensitive TB.

**Table 1:** Table of participants

Characteristics of participants	Distribution
Sex	
Male	47 (59%)
Female	33 (41%)
Age	
Age range	19–75
Self-reported severity of TB symptoms	
Non	3 (4%)
Mild	3 (4%)
Moderate	66 (82%)
Severe	8 (10%)
Number of TB episodes	
1	63 (79%)
2	15 (19%)
3	2 (2%)
Co-morbidity	
Self-Reported living with HIV	37 (46%)
Self-Reported symptoms of mental distress	61 (76%)
Employment	
Formal	17 (21%)
Informal	53 (66%)
Unemployed	10 (13%)

### Summary of mental distress characteristics

Most participants (61/80) shared that they had experienced some form of mental distress during their TB investigation and treatment period. The reported symptoms included feelings of shame and guilt, reduced self-worth, hopelessness, and disengagement from activities that once provided pleasure. For some participants, these feelings were heightened during TB investigation and early on in treatment and subsided once morbidity from TB and side effects of the TB medication improved. The severity of these symptoms also varied. Some participants reported mild distress that did not disrupt their daily lives or ability to adhere to their TB medication. Other participants reported more severe symptoms of distress, for example, 12/80 participants shared that they had suicidal ideation and thoughts of self-harm during their time on treatment and some admitted that these feelings made it challenging to adhere to their medication. No patterns emerged by age. Women were more likely to report their inability to pursue their care giving roles as a cause of distress while men were more likely to be distressed by the disruptions to their role as financial providers for their families. Further analysis that quantitatively compared those with reported mental distress (n = 61) to those without (n = 29) did not reveal any clear pattern in the two groups. However, focusing on the 12/80 who shared suicidal ideation, it emerged that a combination of socio-economic stresses at individual, household and health system levels converge alongside having TB that could contribute to this extreme manifestation of mental distress. The following two case-studies illustrate these multi-layered and intersecting stresses, with the economic stress of poverty often being the most powerful driver.

Case Study 1, Z5 transcript 5: [Married man of unknown age, lived with his wife and parents during his TB treatment. He is HIV positive and had two episodes of TB, the first was in 2016, when he worked as a manual laborer at a cement factory, the second was in 2018 when he worked as a miner. In both incidents he was fired from his job due to his TB diagnosis. He incurred TB investigation costs (transport and Xray) and was evicted from his house because he was unable to pay rent. He sold household items (stove) to try and meet his costs. His second TB diagnosis was delayed for 4 months due to poor medical investigation at the clinic, leading to severe TB morbidity (was hospitalized for one and a half months). He considered stopping his medication due to the side effects. He experienced TB stigma in form of being treated rudely by health personnel, friends avoiding him and being fired from his jobs. He struggled to cope with his wife’s suffering which he attributed to him losing his job and house due to his diagnosis. His wife and mother provided emotional support through his treatment period. He did not receive financial support from family but did mention the doctor gave him money sometimes.]

Case Study 2, Z3 transcript 4: [30–35-year-old single woman, with 2 children. She works as a marketeer. She had recently cared for relatives who had TB. Her TB diagnosis was delayed due to poor medical investigation, and she subsequently developed severe TB symptoms (was hospitalized for one month). During her hospital stay it was also discovered that she was HIV positive. She stopped working and moved in with her sister. She thought she would die and worried about who would take care of her children if she did. She contemplated defaulting on her medication but was encouraged by nurses and her sister to continue. Her sister was very supportive, escorted her to collect her medication and nursed her through her illness. Neighbors and friends also provided financial and emotional support. She did not experience any stigma but was embarrassed by her diagnosis and worried that people were talking about her. She believed TB ran in the family or was acquired through acts that were deemed to be immoral].

### Individual level drivers of mental distress

At individual level, drivers of mental distress included morbidity and pain (77/80), bodily changes and accompanying involuntary disclosure (43/80), fear of death (10/80), HIV co-infection (37/80), and culpability (29/80).

#### Morbidity and pain

TB took a toll on patients’ bodies. The symptoms of TB were a major source of mental distress to participants who described them as painful and immobilizing.

“I did not feel good. I used to sleep one side, I felt like water was boiling on the other side when I slept. I did not sleep without taking painkillers.” (40–45-year-old woman, Z2)

The majority (77/80) of the participants reported experiencing some level of physical morbidity from TB and 8/80 reported morbidity that was so severe that they were unable to walk or bathe themselves. All the participants who reported severe morbidity also reported mental distress symptoms. For some of these participants, their mental distress was so severe that they had suicidal ideation during their TB treatment period. On the other hand, six of the seven participants who reported little or mild morbidity resulting from TB reported no mental distress, with only one reporting very mild mental distress upon hearing their diagnosis.

#### Bodily changes and involuntary disclosure

Participants highlighted that TB symptoms caused visible changes to their bodies. The most notable changes were weight loss and changes in skin appearance which 43/80 participants described. These changes left patients feeling like *“they were not themselves”* because of how different they looked. Some participants reported feeling embarrassed by the way they looked when they had TB and some feared that these changes in their appearance led to unintended disclosure of their diagnosis.

“.. I am light in complexion, but my skin became extremely dark which clearly showed that I was not well” (20–25-year-old woman, Z7)

#### Fear of death

Participants’ reflections of their TB diagnosis were poignantly linked to death. The severity of the TB symptoms that participants experienced led some to think they would die from the disease.

“When one has TB the only thing they think is that they are going to die.” (40–45-year-old man, Z12)

Other participants knew someone who had died from TB, and in some cases, this was someone close to them such as a spouse or sibling who they nursed through the illness. For 10/80 participants, the fear of death was accompanied by a deep and persistent sense of worry that their children would *“suffer”* if they died. There was also a sense of guilt and self-blame about bringing *“suffering”* to their children and family through *“exposing”* them to TB.

“… I would be sitting and look at the children and think, so just like that my children will begin to suffer, I have exposed my children to suffering because I have contracted TB….” (25-30-year-old man, Z7)

#### HIV co-infection

Many participants highlighted the assumption that being diagnosed with TB was an automatic indication of HIV diagnosis and this was very distressing for some participants mainly due to the stigma associated with HIV in this setting. An HIV diagnosis was sometimes described with moral undertones, exemplified by HIV negative participants’ description themselves as being “*correct,”* or *“clean”* or *“never having indulged in bad activities*” when talking about their HIV status. The association between TB and HIV was source of discrimination for some participants, as illustrated by the following:

“My family segregated me because they thought that since I had TB, definitely I was also HIV positive.” (25-30-year-old man, Z2)

For some of the 37/80 participants who shared that they were living with HIV, the co-infection added additional health and emotional challenges, 30/37 of them reported having mental distress symptoms with six reporting suicidal ideas. Many described the experience of the co-morbidity with strong negative emotions, such as feelings of depression and hopelessness. However, half of the participants who reported being suicidal were HIV-negative, and it was thus other individual stresses that underlay their mental health distress.

#### Daily medication regimes

Participants mentioned that their TB symptoms abated after a couple of months of taking their TB medication except for, the challenges they faced with taking the medication that added an emotional burden for some participants. Reported challenges included large size of the tablets, long treatment regimen duration (usually a minimum of six months), debilitating side effects of the medication, and the frequent trips to the health centre to collect medication. These challenges were even more pronounced for participants who were managing other co-morbidities, most commonly HIV. Some of the participants that reported being suicidal mentioned having challenges with adherence and needing to be encouraged by close family to continue. Indeed, one participant expressed that death was at some point a more attractive option than adhering to her medication.

“Yes, I thought it was better to die, so that I stop taking drugs.” (20–25-year-old woman, Z12)

#### Culpability

Popular community beliefs about TB that many participants shared were sometimes inaccurate and often a source of self-blame for their diagnosis. Participants mentioned beliefs that TB was contracted through excessive drinking or smoking, “promiscuous” ‘sexual behaviour (a colloquial expression implying moral judgement about multiple sexual partners or sexual contact with a woman who had had an abortion). These popular assumptions serve as a driver of TB stigma, increased feelings of shame, and reduced self-worth amongst participants. Furthermore, participants shared feelings of guilt for contracting TB, believing it was brought about by some morally unacceptable behaviour. The participant below described self-perceived culpability of contracting TB because of their own “*careless”* smoking:

“I: But did you at some point think that maybe it is your fault for getting TB?

R: Yes, because of my carelessness.

I: Why did you think like that?

R: Because of my smoking of cigarettes”

(30–35-year-old man, Z8)

### Social and economic household level drivers of mental distress

The socio-economic household circumstances of participants that contributed to mental distress include income loss (51/80), depending on others, isolation and/or stigma (35/80).

#### Income loss

Morbidity associated with TB resulted in job or income loss for most working participants, as they became too ill to carry out their tasks. The majority of participants (53/80) worked in the informal sector, the men mainly worked as casual labourers, illegal miners, mechanics or entrepreneurs, while women were more likely to be domestic workers, hairdressers or marketers. The nature of informal work meant their contribution to household living depended on them going out to work, and for these participants, this meant they could no longer make a living whilst they were ill.

A minority of participants (17/80) were in formal employment. Women were either employed as cashiers and office orderlies while men either worked in construction, artisans, the mines or as security guards. Only four of those in formal employment were granted sick leave during the time they were on treatment, and another four reported being fired on medical grounds. Some participants opted to hide their diagnosis from their employers all together. Participants often resumed income generating activities around three to four months after starting treatment, as they begun to feel better. One participant mentioned that he was unable to work as much as he did before having TB and another relayed how the days that he collected medication undermined his business. A smaller number did not manage to work at all during treatment, as detailed in the quote below.

“I was fired from work. I stayed for eight months without working and I no longer work…….I want to go back to work so that I can help my children go to school.” (45–50-year-old man Z2)

The quote above also highlights that TB can undermine educational attendance of children and TB patients; one young woman participant stopped attending school because of having TB and other participants spoke of children struggling to attend school.

Some participants reported that their households sold household items, borrowed food and used savings to get by during the period of having TB and financial support from close family (parents, spouse, siblings) was reported by 16 participants. A few participants also reported financial support from neighbours, friends and church members. No participant mentioned receiving financial assistance from the government. The economic stress of having TB is detailed below.

“TB worries mean there is no food at home because you can’t provide for the family and there will be hunger, you will fail to pay school fees for the children as well as rentals.” (40-45-year-old man Z10)

This was particularly distressing for male participants who tied their ability to earn and take care of their dependants to their identity as a man and expressed that being unable to do this often led to feelings of reduced self-worth and shame.

“….the time I was ill I had worries because I was not able to work so that I earn. They used to bring food for me and that was not good for a married man with children.”

(35–40-year-old man, Z2)

#### Isolation and stigma

Isolation was a common experience during TB treatment and was a concept strongly tied to mental distress during the treatment period. The isolation was either voluntary or forced. Voluntary isolation was practiced by some participants for two main reasons, the first being fear of infecting others with TB, as the participant below highlights.

“When I had TB I stopped going to school or playing with my friends so that I would not infect them…” (20–25-year-old woman, Z5)

One man participant moved household to protect his children from TB, going to stay with his sister during TB treatment. Participants also isolated themselves to avoid being stigmatised. For example, the man below detailed how he avoided people when he went to church by sitting alone at the back bench, and further explained why being away from people was necessary for him.

“People used to laugh when you have TB, so it was very difficult to be found with friends.” (40–45-year-old man, Z8)

However, a notable number of participants explained that isolation was imposed on them by others (often members of their households) through the use of separate cutlery, bedding and enforced social distancing. These experiences magnified aspects of mental distress as participants revealed feelings of shame and reduced self-worth and in some cases, feeling less than human, as is the example in the quote below where the use of separate cutlery made this participant feel *‘like a dog’*.

“R: I felt ashamed of myself because when people know that you have TB they feel like you are something else

I: Something else like what?

R: I don’t know, like dog or I don’t know…. The way some people decide to get a specific plate, spoon, and cup for a TB patient to use alone doesn’t make a person feel free.”

(25–30-year-old-man, Z7)

### Health system challenges

The health system challenges that that contributed to participants mental distress include poor health investigation (40/80), negative attitudes of health staff, and stock out of TB medication and consumables**.**

#### Health investigations at local clinics

There was often a significant delay in being diagnosed with TB, often at least two to three months in part due to inefficiency in the health system. This was the case for close to half the participants who were misdiagnosed and or not adequately evaluated by medical staff at local clinics. In many cases participants were given antibiotics and painkillers without medical investigations being carried out. This delay could prolong morbidity and increase anxiety as health continued to deteriorate despite frequent visits to the health centres. This is illustrated by the following participant:

“I described how I was feeling to the health personnel at the clinic and I was then given medicine. They gave me Amoxil and Panadol. I went back to the clinic and they changed my medication to Anthramycin and Aspirin. I felt better for a while. I went there again and was given an injection with Anthramycin and Panadol. I stayed for a while yet the disease was becoming worse and pain inside were increasing until at last I started feeling eaten up and my voice became weak, my energy level reduced and my heart felt like it didn’t have a place to stay,” (40-45-year-old woman, Z8)

Many of these participants were only properly diagnosed after being referred to the nearest tertiary general hospitals. The following participant notes that she would have most likely died due to lack of proper investigation had she not moved from her local clinic to the general hospital in her area.

“R: I would have died at XX clinic

I: Why do you say this?

R: …the time I was ill, and I used to come here (clinic), and would tell them that the cough is not stopping, however, they would just give me Amoxil. They didn’t check me to know what was causing the cough.” (Unknown-age woman, Z8)

#### Health workers attitudes

Negative attitude by health staff was also cited as a source of distress for participants. Some participants noted that some nurses would ignore them and were rude, when providing them with services. Altercations between health staff and patients sometimes resulted in withholding of care and medication as was the case for the participant below.

“They (nurses) have to learn to care for the patients. There is a nurse who used to shout at me and she even threw away my registration book. She told me to go back to XXX Clinic where I was getting treatment initially. I asked her why we were getting medication at 11:00 hours when we were getting at 07:00 hours at the other clinic. She told me to go back to XXX Clinic if I was not happy and she even refused to give me medication that day. Luckily, I was called by a certain doctor who told me that the nurse was not supposed to do that and he gave me the medication.” (30-35-year-old woman, Z2)

#### Stock out of TB medication and consumables

Other distressing bottlenecks that participants noted included lack of equipment and consumables that hindered the TB investigation process. Additionally, one participant highlighted that sometimes the health facility she collected her medication from did not always have it available.

“I had worries because I felt I would die. The TB medicine would run out a lot (at the clinic).” (20–25-year-old woman, Z5).

## Discussion

TB patients experienced mental distress during their time on treatment. Mental distress expressed by participants was caused by individual, economic, social and health system level factors, highlighting the need for multilevel solutions to address mental health needs of TB patients during TB treatment and investigation. Symptoms of mental distress recalled by participants during their time on TB treatment were often synonymous with those of depression and anxiety disorders. This analysis highlights the patient experiences of social drivers of mental distress during TB investigation and treatment and therefore emphasises the role of social context in the mental health experiences of TB patients in this population.

Similar to findings from other settings, participants reported physical morbidity resulting from TB as one of the sources of their distress [36, 37]. Symptoms of TB, particularly pain, wasting, and other changes to participants’ appearances were a major source of concern. Physical morbidity as a contributor to mental distress is not unique to TB: a recent meta-analysis focused on developing and emerging countries found a pooled prevalence of 36.6% of mental disorders in individuals with chronic physical illness [38]. Furthermore CMD such as depression increase the level of morbidity and pain experienced by individuals with chronic illnesses [39]. For some participants, TB presented a substatial threat to their lives and the morbidity they experienced brought them to a point of existential reflection. The potential finality of life was understandably distressing for participants, particularly for those who had witnessed a loved one die from TB or those who feared for the wellbieng of their children. Fear is a natural response to a life threatening illness and it could aggrivate morbidity [40]. Fear of death, and fear of the social and economic consequences of TB was also reported in qualitative studies from other settings inclduing India [41], Vietnam [42], Parkistan [43] and China [44]. Furthermore, TB patients are likely to be experiencing an amalgamation of personal lossess resulting from their morbidity, some of these losses that came up in our findings included: a loss of independence, bodily image, physical functions, self worth, and potential loss of future. Similar algamation of losses have also been documented with other chronic illness [40].

Co-morbidity was another individual level driver of distress. In addition to TB, 46% of our participants also self-reported living with HIV, another chronic condition that could have debilitating implications on an individual’s health if not well managed [45]. HIV is one of the leading drivers of the TB epidemic in Zambia. In 2019, Zambia had an estimated HIV prevalence of 11.5% amongst adults aged 15–49 [46]. Global indicators suggest that Zambia has one of the highest estimated numbers of incident TB cases among people living with HIV globally [13]. Similar to our sample, 47% of TB patients in Zambia were co-infected with HIV in 2019 and co-infected individuals accounted for 62% TB related deaths that occurred in that year [13]. The literature highlights that an HIV co-infection significantly increases the risk of experiencing mental distress during a TB episode [37]. A study conducted in Ethiopia comparing the mental health status of TB/HIV coinfected patients to non-co-infected patients found coinfected patients had a significantly higher prevalence of mental illness (63.7%) compared to non-co-infected patients (46.7%) [16]. These findings are echoed in our data, highlighting the urgency of mental health services for co-infected individuals.

At societal level stigma interfacing with TB that was cited as a source of distress for participants. There were some accounts of discrimination, such as gossip, job loss, and being treated rudely by health workers however, the most common forms of stigma reported were anticipated and internalised stigma. TB is a highly stigmatized condition in Zambia, and its implications have been thoroughly documented [35, 47, 48]. Studies from different setting highlight the link between TB stigma and poor mental health, for example a qualitative study from India found depression, anxiety and suicidal ideation were common outcomes of TB stigma [49]. In Lesotho participants who reported internalised, anticipated, and experienced stigma were 2.4, 1.7 and 2.9 times more likely to have moderate to server depressive symptoms than TB patients that did not report these stigma measures [50]. While a global systematic review consisting of 25 papers found TB patients with perceived TB stigma were 4 times more likely to develop depression than those without perceived stigma [51]. Participants in our study highlighted that TB symptoms often resulted in a different form of stigma, namely TB-HIV stigma. Bond and Nyblade [11] argue that this form of disease stigma is present in settings with high HIV prevalence such as Zambia due to the interconnectedness of TB and HIV in people’s perceptions and understanding the conditions. Additionally, TB patients dealing with a mental health condition such as depression or anxiety could also experience mental health stigma which is also very prevalent in this setting [52, 53].

The economic implications of a TB episode were also highlighted as sources of distress by participants in the study. TB affected participants’ ability to engage in income generating activities thereby often increasing the economic vulnerability of participants and their households. This increase in economic vulnerability is occurring in a setting with relatively high levels of poverty. The TB epidemic in Zambia is concentrated amongst lower socio-economic brackets particularly in urban settings which are exposed to the structural drivers of TB such as poor nutrition and overcrowded and lower quality housing which create a suitable environment for TB transmission and disease progression [54]. Independently of TB, poverty also serves as a driver of mental distress, according to a 2020 systematic review, within each given setting individuals in the lowest income brackets are 1.5 to 3 times more likely to experience CMD than those in the highest income brackets [55]. Therefore, in high TB burdened settings people living in poverty are at high risk of independently and jointly developing TB and mental ill health due to shared risk factors between two. Concurrently TB and mental ill health can both independently and jointly lead to impoverishment due to reduced productivity levels as a result of morbidity and the direct and indirect health related costs associated with both conditions [56]. For example, our data revealed that for 51/80 participants, TB illness resulted in income loss due to an inability to work and 48/80 reported indirect costs resulting from TB investigation and treatment.

Lastly participants also highlighted health system level drivers of distress similar to those documented in the literature from the region [57]. One of these health system failures included lack of adequate investigation at health facilities which was reported by half of our participants. There is evidence from a 2013–2014 TB prevalence survey conducted in Zambia supporting our findings, 49.7% (80/161) of the symptomatic participants diagnosed with TB during the survey had previously sought care for their symptoms at a health facility and were not screened and subsequently not diagnosed for TB highlighting the magnitude of missed opportunity for TB diagnosis due to poor investigation in this setting [20]. In a 2022 global systematic review four studies indicated that symptom screening was not conducted for 33–96% of patients with TB symptoms while only a median of 38% of participants with TB symptoms were offered a diagnostic investigation [58]. This has implications for both the individual and the community. At individual level delayed diagnosis leads to increased severity of the disease, higher investigation and treatment costs, and poorer treatment outcomes which all ultimately contribute to mental distress in individuals with TB [37].

## Recommendations

Based on our finding we propose the following multi-layered recommendations to improve mental health and TB treatment outcomes of TB patients in this setting.

Individual drivers of mental distress in TB patients, including morbidity and fear of death, need to be acknowledged by TB health care workers. Therefore, in addition to treating and educating patients on the curability of TB, health workers need to be sensitive to patients experiences of the illness, including the realities of the potential losses that patients have encountered due to morbidity from the illness. This calls for a more patient centered approach that transcends biomedical manifestation of TB by accounting for the multi-faceted ways in which TB affect patients lives. Patient centered care has taken on various models. A 2012 global systematic review of 32 studies focused on patient-centered care in chronic disease management highlights integral components of patient centered care including: acknowledging the patient’s situation; legitimizing the illness experience; and offering realistic hope [59]. The acknowledgement of patient-centered care in TB treatment has resulted in it forming an integral part of the WHO first pillar of the post-2015 TB strategy [60]. As part of patient centered care the WHO recommends that all TB patients should receive educational, emotional, and economic support during TB investigation and treatment to prevent treatment default [60]. Patient centered care has in some cases been shown to improve patient wellbeing including mental health outcomes [61].

At societal level our findings reveal stigma as a major driver of distress for TB patients. It is therefore vital to tackle the multiple stigmas associated with mental ill health, TB and HIV at household, community, and health setting level in Zambia. More research is required to create context sensitive interventions that would account for the structural and contextual factors underlying the intersecting stigmas. Policy makers in Zambia can utilize the health-related stigma framework created by Stangl et al. (2019) as a guiding tool to creating research and intervention around intersecting health stigmas in the Zambian context. The benefit of this framework is that it acknowledges and addresses parallels in drivers and consequences of stigma across different conditions which is a shift from the existing stigma frameworks that typically take a siloed approach thus focusing on each health condition in isolation.

Economic drivers of distress include the income loss due to indirect costs during TB investigation and treatment, coupled with a significant reduction in productivity, often surmounting to catastrophic health costs for individuals with TB and their families. None of the participants in our study were in receipt of welfare state, highlighting the need for safeguards against job loss resulting from morbidity of TB, in addition to provision of social protection schemes for economically vulnerable TB patients. There are several laws in Zambia promoting these safeguards, but our findings indicate that these needs improve enforcement and accountability mechanisms. Economic stressors among TB patients are not new to this context. Previous research has highlighted the importance of transport and food aid for TB patients and people living with HIV on ART in Zambia [62]. Social Protection schemes could potentially alleviate one of the major drivers of mental distress in TB patients, as well as improve TB treatment outcomes for individuals, for example a 2018 global systematic review of five observational studies, with a total of 21,976 participants found that TB patients that were receiving socioeconomic support were 1.8 times more likely to have positive clinical outcomes that TB patients that did not receive socioeconomic support [63]. However, more research is required to guide the most appropriate form and implementation of social protection for this specific context.

At health systems level, our data revealed a dearth of mental health support services, despite the overwhelming need for them by our study participants. Mental health services are typically provided in centralised psychiatric hospitals located in the provisional headquarters across the country [8], with the exception of HIV counselling services, which have proliferated in Zambia since the 1980s [64]. Originally these followed a home based care model, aimed to provide psychological support to AIDS patients and their families [64]. With the increased availability of ART, HIV counselling services have evolved to facility based services with an emphasis on adherence counselling, and advice on sexual and reproductive behaviour of people living with HIV [64]. Similarly, TB treatment supporters also provide advice and encouragement to TB patients with a focus on adherence support to TB patients. To address the dearth of mental health support services for TB patients, it may be appropriate to train TB treatment supporters to screen, treat and/or refer their patients with comorbid mental illness. The WHO Mental Health Gap Action Programme (mhGAP) provides guidance on how this can be achieved through a task shifting approach, where non-mental health specialists in the primary health care setting are trained to identify CMD and provide low-intensity intervention, using evidence-based techniques [65]. In this model, patients with severe symptoms (such as suicidal ideation) are referred to specialists for more intensive support. Government commitment in providing adequate and regular training, management and sufficient remuneration is essential for such a model to be successful [66, 67].

## Strengths and limitations

This is the first study to explore patient perspectives on drivers of mental distress during TB investigation and treatment in this context. The major strength of the study was the sample size of participants which allowed for exploration of diverse experiences and perspectives from a sample with national level representation.

The weaknesses of the study include recall and selection bias. These limitations arise from this research being embedded in a larger TREATS study that aimed to explore experiences of TB patients who were diagnosed through standard of care, and those diagnosed through active case finding through the PopART study. The sample was therefore restricted to those who were diagnosed and completed treatment between September 2016 and November 2017 when the PopART intervention was still being conducted. Therefore, participants had to recall their time on TB treatment during data collection which occurred from October 2018 to February 2019. Additionally, our sample of participants had all completed TB treatment, and we are therefore unable to explore the mental health experiences of individuals who defaulted on their treatment. Furthermore, our analysis was unable to stratify based on time since TB treatment completion and mental health status prior to TB diagnosis. Finally, generalizability of our findings is limited to settings like Zambia, that is, high TB burdened and low to middle income countries.

## Conclusion

Mental distress was pervasive among this group of TB patients from eight urban Zambian and geographically distinct communities and has implications on their treatment experience. Context plays a key role Mental distress experiences among TB patients in Zambia. The findings illustrate that mental distress during TB is driven by multi-layered and intersecting stresses, with the economic stress of poverty often being the most powerful driver. Measures are urgently needed to support TB patients during the investigation and treatment phase, including increased availability of mental health services, better social security safety nets during TB treatment, and interventions targeting TB, HIV and mental health stigma.

## Supplementary Information

**Additional file 1.** Tb Patient Individual Interview Guide. Qualitative data of TB patients TB investigation and treatment experiences in eight Zambian communities.

## Data Availability

The data used or analysed during the current study are available from the corresponding author on reasonable request.

## Abbreviations

CHiPs: Community HIV care Providers
CMD: Common mental health disorders
IDIs: In-depth interviews
HIV: Human Immunodeficiency Virus
LMIC: Low- and Middle-Income Countries
LSHTM: London School of Hygiene and Tropical Medicine
PopART: Population Effects of Antiretroviral Therapy to Reduce HIV Transmission
TB: Tuberculosis
TREATS: Tuberculosis Reduction through Expanded Antiretroviral Treatment and Screening for active TB trial
WHO: World Health Organization
