# Supplementary material for: Qualitative study of patient experiences of mental distress during TB investigation and treatment in Zambia
Source: BMC Psychol. 2022 Jul 19;10:179. doi: 10.1186/s40359-022-00881-x (PMC9295264; doi:10.1186/s40359-022-00881-x)
Supplement: Supplementary file 1 — Additional file 1. Tb Patient Individual Interview Guide. Qualitative data of TB patients TB investigation and treatment experiences in eight Zambian communities. [file 40359_2022_881_MOESM1_ESM.docx]

TREATS Qualitative Work Package- Tb Patient Individual Interview Guide

**TB PATIENT INDIVIDUAL INTERVIEW GUIDE**

*Objective: To understand the experiences of TB patients and any influence of PopART on these experiences, including the influence of CHiPs.*

*Expected time needed: 1 – 2 Hours*

Date activity conducted: ___________________________

Place conducted: _____________________________

Time period: _____________________________

*Materials Needed: map of the community, map of the clinic, coloured crayons (red, blue, green), informed consent forms, audio-recorder, notebook, pen, pencil*

*Form of data recording: (1) Audio-recording of all talk from “Preamble” to “Closing” depending on the tool used. (2) Notes of key points per topic area handwritten by the facilitator into a printed copy of this document. (4) Handwritten notes by participants during the course of the discussion.*

*Preamble* (to be read by facilitator): Today is the (insert date [day xx^th^ Xxx xxxx]) and it is (insert time XX: XX). This is a discussion with a TB patient (community member). Thank you for your time. As we have explained, we are conducting this interview as part of the social science research of the TREATS study. For this interaction we would like to find out more about your life. All information collected here will be reported anonymously. I remind you that we are audio recording this discussion and ask that you speak loudly and clearly. As the facilitator I will also be taking some notes. Do you have any questions before we begin?

**Participant details:**

Name of Interviewer(s): ____________________________

Date: ___________ Time Period -Start: _____________ Finish: _____________

Location [District, Province]: ­­­­­­­­­­___________________________

Place interview conducted: _____________________________

Interviewee’s Name: _____________________ Position:____________________

Age [in years]: _____________ Sex: ____________

**INTRODUCTORY QUESTIONS**

1. I understand you have/had TB. Could you tell me how long you have been on TB treatment? Is this your first time to have TB?
2. How long have you lived in _____________?
3. Where did you live before you moved to ______________?
4. What was your main reason for coming to__________?

- Had family here
- Had friends here
- Work
- Displaced
- Married here
- Had family/friends in area
- Studies/education
- Seeking treatment for current illness
- Housing
- Other……….

1. Before you developed TB, what did/do you do to contribute to household living?
2. Who do you live with at the moment? *Collect details of household members and relationship to patient.*

**PERCEPTIONS AND TRANSMISSION**

1. Did you consider yourself at risk of getting TB?
2. Where do you think you got your TB? Within this community or outside?
3. Looking at the map of this community *[map of the community]*, could you let us know firstly where you spend time on a regular/daily basis (and with whom)?
4. In your opinion, would any of these places (and/or people) have exposed you to TB?

- *Probe: Places: Bars/shebeens/taverns, clinic, churches, video/games clubs, hair salons, schools, houses (which kind), minibuses/buses/taxis*
- *Probe: What is it about these places? E.g. poor ventilation, small/blocked/no windows, overcrowding, small rooms, other TB patients, sexual behaviour, smoking, charcoal stoves inside houses, sharing utensils, prolonged time in that space.*

1. How did you feel when you were diagnosed with TB?
2. Do you fear that you might pass your disease to others in your family? [PROBE ON HOW LONG S/HE THINKS S/HE IS/REMAINS INFECTIOUS TO OTHERS]
3. Would you kindly tell us the precautions you take as a TB patient to prevent transmission of TB to others?
4. What precautions do your family members take to cut down transmission of TB in the house?

**TREATMENT SEEKING**

We would now like to talk about where you looked for treatment for your TB in this community. Could you think back to the time that you first had symptoms and talk about everything that happened until you finally got diagnosed with TB? *It is critical to allow any contact with CHiPs to emerge in this sequence of health seeking, alongside other treatment options. If it doesn’t emerge, but TB patients were selected on basis of being diagnosed through CHiPs, then probe for it once you have allowed the participant to discuss all the options and decides they wish to.*

**USING CHART, LEAD PARTICIPANT WITH THE FOLLOWING QUESTIONS:**

- When you first knew you were sick, what did you do to treat your symptoms?

**USING NETWORK CHART**

**FOR EACH TREATMENT SOURCE MENTIONED ASK:**

- How did you know about it?
- What happened there?
- What treatment were you given?
- How long was the course of treatment?
- How much did it cost?
- Did they refer you elsewhere?
- Of these places/people, are there any that you feel uneasy about?

1. If contacted by CHiPs ask them to recall in some detail what happened during the interaction with CHiPs. Probes include:

- initial contact with the household,
- TB screening questions,
- producing sputum and giving sputum to the CHiPs,
- going to the health facility with sputum,
- waiting and receiving results,
- any follow up and support from CHiPs
- ask about differences between CHiPs and other volunteers, including TB treatment supporters.

1. How long did it take you to get diagnosed with TB?
2. Did you decide on your own or a household member advised you to seek treatment for your symptoms

- **If seeking treatment was delayed for more than two weeks, probe reasons for delay** (PROBE: fear of diagnosis, treatment course or completion, stigmatisation, affordability, accessibility of health centre)

1. Where do you receive your treatment from (clinic, DOTS in the community, hospital?).

- How do you get there?
- How long does it take you to get there?
- How often do you go there?
- When were you last there?
- About how long were you there last time (record hours)?
- How much does it cost to get there?

1. Looking at the map of the clinic you are attending as a TB patient *[map of the clinic]*, could you explain:

- Where you went in the clinic when you first went there with TB symptoms? *Probe for role of CHiPs in navigating the clinic.*
- How were you treated?
- Where did you go within the clinic now you are diagnosed with TB symptoms?
- Using these three colours, could you colour places within the clinic thinking about:
  - Places that make you feel comfortable (blue)
  - Places that make you feel uncomfortable (green)
  - Places where people talk badly about TB patients (red)

1. Do you worry about going to the place where you get your treatment? What worries you?
2. What is your opinion of the treatment that you receive at the health centre/clinic, both in the TB clinic and outside of it?

**TB TREATMENT**

1. Can I ask what medication you are taking?

- Probe for details about medication – collection (how, where, who), what medication, side effects, other problems, number of pills or injections a day, supervision of treatment.

1. Do you ever hide the fact that you are on TB treatment?
2. Since you started taking TB treatment, is there any period when you stopped taking medication?

- Was there any period when you considered stopping medication?

1. Have you or do you take any other therapy? For example, immune boosters, herbs, faith healing, witchfinding?
2. Do you believe you will be cured of TB?

**IMPACT OF TB**

1. Have you been admitted to hospital for your illness, how long? Or cared for at home?
2. How has TB affected your:

**-** Body?

**-** Daily activities? (Perform daily tasks, personal care)

**-** Participation in life? (Probe for ability to work and earn, provide)

1. Has TB stopped you doing what you should be doing as a man/woman? Does this worry you? (probe for self-criticism from self or others)
2. Do you have any ideas what might reduce stress and worry for men and women with TB?
3. If having TB worries you, how does it compare with other worries? Can you name any of the other worries?
4. Were you able to talk about your worries to anyone in your family or health care worker or others?
5. Are you able to say what has helped reduce your worries? (If that is the case) or made your worries worse?
6. How has TB affected your relations with your friends, relatives not living with you, neighbours and other people in the community?

1. How has having TB affected your relations with household members?

**TB STIGMA**

1. Do you feel that people act nervously around you because you have TB? Probe: Who? Why? An example of this?
2. Do you think that people feel pity for you because you have TB? Probe: in what way (how do you recognise this?)? Who? Why? Example?
3. Do people try to stay away from you because you have TB? Probe: in what way (how do you recognise this?)? Who? Why? Example?
4. Do you feel isolated because you have TB? Probe for physical isolation (with examples) and social isolation (with examples) and emotional isolation (with examples)
5. Do people think it is your own fault that you developed TB?
6. Do you think it is your own fault that you developed TB?
7. Do you feel ashamed about having TB?
8. Do people feel angry towards you because you have TB? Probe: Who? Why? An example of this?
9. Do people gossip about you having TB? Please give examples.
10. Do people talk badly about you to your face because you have TB? Please give examples.
11. Since falling ill with TB, have you experienced any direct discrimination because of your TB? For example, losing a job, being thrown out of a house, being excluded from church/market/school/social events, divorce/separation
12. In your opinion, is TB stigmatised? What can you and others do about reducing TB stigma?

**SUPPORT**

1. Who have you shared your TB diagnosis with:

- Inside the household?
- Outside the household?

1. What kind of support do you get from your family (*Probe: emotional, financial, practical*)?
2. Was there a time when you felt very sick and unable to take care of yourself?
3. Who in the household helps you in times of need – accompanied to the health centre, household member fetch medication for you, and preparation of food, personal hygiene? *Probe for identity of primary caregiver.*
4. Outside the household where do you get support in times of need?

**TB TREATMENT AND FOOD CONSUMPTION**

1. Do you have any problems taking your TB medication?
2. Is it true that TB medication makes you hungry?
3. How is the hunger linked to TB medication different from the hunger you felt before you were sick with TB?
4. Are you able to get food every time you feel hungry? Explain how you manage to get food.

- Use cash savings to buy food
- Defer payment for other household needs (what)
- Sell household assets
- Borrow money to buy food (from whom)
- Borrow food from the local shop (credit)
- Receive remittances (in cash or kind – from whom. If in kind what was received)
- Sustain hunger

1. What kinds of food were you told to eat or not to eat because of your illness? By whom?

1. If you ask for special food, how do others in the household react?

**TB/HIV CO-INFECTION**

1. Have you been tested for HIV?
2. Would you mind telling me your result?
3. If HIV-negative, did you worry that you might have HIV when you were diagnosed with TB?
4. If HIV-positive, would you mind telling me about having HIV and TB at the same time?

- *Probe: If co-infected with HIV, how does TB compare to HIV? How long have you known you had HIV? Are you on ART? If on ART, how does it feel taking two types of medication at once?*

1. Do people in the community link TB to HIV?
2. Which is worse in your experience – TB stigma or HIV stigma? Has this changed over time?

**CLOSING QUESTIONS**

1. How do you feel about your own future at the moment?
2. Thinking back and thinking about this community and TB in this community, do you think TB is now better or worse than it used to be?
3. If you had to improve the situation of people like you (with TB), what would you do? *Probe: food security, clinic services, stigma reduction*, *housing type*
4. Finally, what do you think is the role of volunteers (like CHiPs or TB treatment supporters) for TB patients? Do you think we need them to do this work on TB?

We have now finished our discussion. Now, are there any questions you would like to ask us? Thank you very much for participating. We really value your time and the information and experiences you have shared with us
